# The challenges of explainable AI in biomedical data science

**DOI:** 10.1186/s12859-021-04368-1

**Published:** 2022-01-20

**Authors:** Henry Han, Xiangrong Liu

**Affiliations:** 1 Department of Computer Science, School of Engineering and Computer Science, Baylor University, Waco, TX 76798 USA; 2 Department of Computer Science and Engineering, Xiamen University, 422 Siming S Rd., Siming District, Xiamen, 361005 China

With the surge of biomedical data science, more and more AI techniques are employed to discover knowledge, unveil latent data behavior, generate new insight, and seek optimal strategies in decision making. Different AI methods have been proposed and developed in almost all different biomedical data science fields that range from drug discovery, electronic medical records (EMRs) data automation, single-cell RNA sequencing, early disease diagnosis, COVID research, and healthcare analytics. The AI methods and systems also generate a massive amount of data or big data that not only bring unpreceded progress in biomedical fields but also new challenges for AI.

One of the key challenges should be the explainability of AI in biomedical data science problem-solving. It refers to that an AI method or system should not only bring good results but also have good interpretability, i.e., let users know why this way is the optimal one rather than the others. The existing AI methods employed in biomedical data science generally lack good explainability and may not create trustworthiness and transparency in usage well. For example, a deep learning model may bring good accuracy in disease diagnosis by analyzing corresponding bioimages, but it can be hard to explain well about the setting of thousands of parameters in the model. It can be possible that some small perturbations of the parameters may generate totally different learning results and challenge the robustness and stability of the deep learning model. Since AI models cannot explain themselves well, it is likely to encounter a high risk to make an incorrect decision making and decrease its trustworthiness and reliability, even if it has the advantage in accuracy, speed, or complicate data relationship revealing.

On the other hand, the AI interpretation issue has been raised almost ten years ago in some subfield of biomedical data science such as bioinformatics. For instance, bioinformaticians found that the gene markers or network markers recommended from an AI disease diagnosis system may not explain themselves, i.e., the identified markers not only cannot apply themselves well in clinical practice, but also those markers that do well in the clinical practice may not be recommended from the AI system [1]. Different methods may generate totally different network markers even under the same dataset. Although disease complexity and high nonlinearity of omics data can be a reason for this, another important reason should be the AI methods employed in the systems lack enough interpretability and transparency, which generate different solutions in a black-box way. Therefore, it is urgent to develop explainable AI (XAI) that acts more transparently to provide practitioners or investigators reliable results along with good interpretations on ‘why it works’ rather than only ‘it works’. Furthermore, biomedical data science may require a higher standard for the AI methods’ interpretability and transparency because of its special subjects and application domains. It can be hard or even dangerous to believe the results from non-transparent AI methods in healthcare, drug discovery, or disease diagnosis because opacity can be harmful and unpredictable.

However, there are challenges in achieving explainable AI in biomedical data science. We mainly address it from AI method customization, nonlinear data, and problem-solving complexity, and learning bias perspectives. First, almost all state-of-the-art AI techniques are not developed for biomedical data. Instead, they are originated from computer vision, image recognition, automated reasoning, cognition, or even statistics. It can be challenging to migrate the existing AI techniques to biomedical data science in an explainable way. The AI methods should be customized or even modified to individual datasets on behalf of good performance and interpretation rather than simply applying them. However, such a customization process may not be achieved in a short period easily, because there is no mature AI theory to guide it and the required degree of explainability can vary with different application domains.

Second, biomedical data science includes various types of massive data that range from sequencing data, high-dimensional omics data, text, EMRs, and bioimage data. The size, nonlinearity, and complexity of the data along with the biologically complicate problems, most of which are disease-related, sometimes force the AI methods to make the trade-off between a good performance and a good explainability. It is likely that good performance cannot be achieved well among a lot of biomedical data science applications. Thus, explainability may not be the top priority from a problem-solving perspective. It is possible that some AI methods with good explainability but a mediocre performance may exist, but they would not be selected by biomedical data scientists due to the consideration of efficiency.

Third, the learning biases created from AI or machine learning methods employed in biomedical data science sometimes prevent the AI methods from providing the minimum interpretations. The learning bias issue refers to the AI results themselves are biased or even totally wrong [2]. The learning bias can be caused by the mismatched interactions between some AI methods and a certain type of data, wrong parameter setting or tuning, imbalanced data, or other more complicated issues, but it may not easily be identified by biomedical data scientists. The learning bias is technically a learning security problem that produces uncontrollable results because of the artifacts in the AI models. The explainable AI should be built upon the assumption that the AI methods can achieve good results and do not have any learning security issues. However, many widely used AI models that range from kernel-based learning, ensemble learning, to deep learning all have or potentially have some learning security issue for certain types of biomedical data. Solving the AI learning security or fixing the learning flaws can be more important than AI explainability for some application domains such as disease diagnosis in translational bioinformatics [3].

Recent research efforts have seen good progress in explainable AI, where rule-based learning, learning process visualization, knowledge-based data representation, human-centered AI-model evaluation etc. are employed to enhance AI explainability [4]. It is  undoubtedly true that the techniques will contribute to the explainable AI in biomedical data science. However, how to overcome the challenges and develop explainable and efficient AI algorithms may need more concerns and efforts in biomedical data science research. It is possible that explainable AI can contribute to enhancing the efficiency and security of AI, but AI explainability should be addressed based on the well-done efficiency and security of customized AI methods developed biomedical applications.

On the other hand, the AI explainability should also have different rigorous metrics to satisfy different needs in biomedical applications. AI explainability should aim at achieving good efficiency and unbiased results in an understandable way to enhance the transparency and trustworthiness of the AI models rather than simply emphasize the users’ understanding. When AI learning efficiency was good enough, the AI learning security issues were clarified and fixed well, and the explainability evaluation metrics were mature enough, then the era of explainable AI would finally come, but it will not come with the same speed for all the biomedical data science domains.
